# The Risk of Developing Diabetes in Association With Long Working Hours Differs by Shift Work Schedules

**DOI:** 10.2188/jea.JE20150155

**Published:** 2016-09-05

**Authors:** Akira Bannai, Eiji Yoshioka, Yasuaki Saijo, Sachiko Sasaki, Reiko Kishi, Akiko Tamakoshi

**Affiliations:** 1 Department of Public Health, Hokkaido University Graduate School of Medicine, Sapporo, Japan; 1 北海道大学大学院医学研究科社会医学講座公衆衛生学分野; 2 Department of Health Science, Asahikawa Medical University, Asahikawa, Hokkaido, Japan; 2 旭川医科大学健康科学講座地域保健疫学分野; 3 Center for Environmental and Health Sciences, Hokkaido University, Sapporo, Japan; 3 北海道大学環境健康科学研究教育センター

**Keywords:** cohort study, occupational health, sex, shift work, working hours, コホート研究, 産業衛生, 性, シフト勤務, 労働時間

## Abstract

**Background:**

The impact of long working hours on diabetes is controversial; however, shift work is known to increase the risk of diabetes. This study aimed to investigate the association between long working hours and diabetes among civil servants in Japan separately by shift work schedules.

**Methods:**

A prospective cohort study was conducted from April 2003 to March 2009. A total of 3195 men aged ≥35 years who underwent an annual health checkup at baseline were analyzed by shift work schedules (2371 non-shift workers and 824 shift workers). Self-reported working hours were categorized as 35–44 and ≥45 hours per week. The incidence of diabetes was confirmed by fasting plasma glucose concentration ≥126 mg/dL and/or self-reported medical diagnosis of diabetes at the annual checkup. A Cox proportional model was used to calculate hazard ratios (HRs) and 95% confidence intervals (CIs) for developing diabetes associated with long working hours.

**Results:**

The median follow-up period of non-shift and shift workers was 5.0 and 4.9 years, respectively. During this period, 138 non-shift workers and 46 shift workers developed diabetes. A decreased HR was found among non-shift workers working ≥45 hours per week (HR 0.84; 95% CI, 0.57–1.24); however, shift workers working ≥45 hours per week had a significantly increased risk of diabetes (HR 2.43; 95% CI, 1.21–5.10) compared with those working 35–44 hours per week. An analysis restricted to non-clerical workers also showed similar results.

**Conclusions:**

The risk of diabetes associated with long working hours differed by shift work schedules.

## Abstract (ja)

**【目的】:**

長時間労働の糖尿病に与える影響は現在までのところ明らかではない。一方、シフト勤務は糖尿病のリスク増加に関連すると指摘されている。そこで本研究では日本の公務員を対象とし、長時間労働と糖尿病の発症の関連をシフト勤務別に検討することを目的とした。

**【対象と方法】:**

本研究は2003年4月から2009年3月にかけて行われた前向きコホート研究である。ベースライン時（2003年4月から2004年3月）に健康診断を受けた35歳以上の男性公務員3195名(非シフト勤務者2371名、シフト勤務者824名)をシフト勤務別に解析した。労働時間は質問票で尋ね、週当たり35-44時間、45時間以上に分類した。糖尿病の発症は年次の健康診断において、空腹時血糖126 mg/dL以上あるいは医師の診断（質問票にて自己申告）で確認した。Cox比例ハザードモデルにて長時間労働に関連した糖尿病発症リスク（ハザード比[HR]と95％信頼区間[95％CI]）を算出した。

**【結果】:**

追跡期間の中央値は非シフト勤務者で5.0年、シフト勤務者では4.9年であった。追跡期間中に非シフト勤務者では138名が、シフト勤務者では46名が糖尿病を発症した。多変量解析の結果、非シフト勤務者では週当たり労働時間35-44時間のものに比較して、45時間以上のものでHRが低下した（HR 0.84 [95%CI 0.57-1.24]）。一方シフト勤務者で週当たり45時間以上働くものでは週当たり35-44時間のものに比較して、有意に増加した糖尿病発症リスクを認めた（HR 2.43 [95%CI 1.21-5.10]）。また全解析対象者のうち、シフト勤務者はそのほとんどが事務員以外の職種で構成されていたため、解析対象者を事務員以外のものに限定して解析したが同様の結果であった。

**【考察】:**

長時間労働に関連した糖尿病発症リスクは、シフト勤務の有無により異なった。

## INTRODUCTION

Long working hours is an important public health concern because of possible harmful effects on human health. A previous systematic review concluded that long working hours is associated with increased risks of coronary heart diseases, anxiety, depressive state, and detrimental sleep conditions.^1^

Regarding working hours, the average annual hours actually worked per worker in Organisation for Economic Co-operation and Development (OECD) countries decreased gradually from 1843 hours in 2000 to 1770 hours in 2013; a similar trend was observed in Japan, where annual hours per worker decreased from 1821 hours in 2000 to 1734 hours in 2013.^2^ However, it was estimated that 22.0% of workers worldwide worked more than 48 hours per week in 2007.^3^ In Japan, the proportion of workers working ≥49 hours per week was 22.9% in 2009 and 21.7% in 2013.^4^ Moreover, that proportion among civil servants in Japan increased from 17.4% in 2000 to 34.2% in 2010.^5^ Thus, it should be recognized that certain workers continue to work long hours.

Diabetes is a common and increasingly prevalent disease worldwide. The global prevalence of diabetes was estimated at 8.3% among adults in 2013, and it was predicted to increase to 10.1% in 2035.^6^ Complications of diabetes (eg, cardiovascular disease, nephropathy, nerve damage, and retinopathy) are major causes of disability, reduced quality of life, and death.^6^

Several studies have investigated the association between long working hours and diabetes^7^^–^^10^; however, the results were limited and inconsistent. From the results of three cohort studies,^7^^–^^9^ Kawakami et al indicated that long working hours significantly increased the risk of diabetes among Japanese male industrial workers.^7^ In contrast, one other study found a significantly decreased risk of diabetes in male office workers working long hours in Japan.^8^ Furthermore, no association was found between working long hours and diabetes among female nurses in the United States.^9^ Finally, a cross-sectional study in Japan that included both sexes showed significantly decreased risks of diabetes associated with long working hours, except for workers with ≥100 hours of overtime per month.^10^

Referring to a previous report,^11^ possible reasons for this inconsistency might include differences in the study design, participant characteristics, categorization of working hours, the definition of diabetes, and the approach to shift work schedules (eg, analyzing separately by shift work schedules or adjusting those as covariates in statistical analysis).

In this study, we focused on the latter factor, because a previous review argued that it is important to deal properly with shift work schedules for clear interpretation,^1^ and a recent meta-analysis concluded that shift work is associated with an increased risk of diabetes.^12^ Based on these facts, we considered that statistical analyses needed to be conducted separately by shift work schedules when investigating the association between long working hours and diabetes. However, no prospective cohort study to date has followed this approach. The purpose of this study was to prospectively explore the association between long working hours and diabetes separately by shift work schedules among civil servants in Japan.

## METHODS

### Study design and participants

This was a prospective cohort study conducted from April 2003 to March 2009 in Sapporo City, Hokkaido Prefecture, Japan. The study population consisted of civil servants aged ≥35 years (total *n* = 10 423; 8229 men and 2194 women) who underwent an annual health checkup between April 2003 and March 2004. Self-administered questionnaires were distributed to them before their health checkup. Those agreeing to participate in this study provided written informed consent and completed the questionnaires, which were then collected during the checkup.

A total of 3962 men and 1051 women consented to participate in this study at baseline (response rate: 48.1% for men and 47.9% for women). Because few women developed diabetes during the study period (*n* = 28), only men were analyzed in this study. For follow-up of at least 1 year, we excluded 176 men aged ≥59 years who would reach retirement within that period. We also excluded 199 men with fasting plasma glucose (FPG) concentration ≥126 mg/dL, and/or who self-reported a diagnosis of diabetes by a medical doctor. In this study, participants with medical histories of angina pectoris (*n* = 25), acute myocardial infarction (*n* = 16), and brain stroke or hemorrhage (*n* = 29) were also excluded.

Working hours were self-reported as hours worked per week. In this study, we considered that participants who worked at least ≥35 hours per week were eligible because their contracted working time was 38.75 hours per week. A total of 88 participants who did not meet this criterion were excluded. Three participants who stated that they worked extremely long working hours (eg, 490 hours per week) were excluded because their responses were considered erroneous. Thirty three participants with missing data for working hours or shift work schedules were also excluded. After the first checkup, 198 men did not undergo the subsequent checkup during the study period. Therefore, the total study sample for analysis comprised 3195 men. The study protocol was approved by the Ethics Committee of Hokkaido University Graduate School of Medicine.

### Working hours, shift work schedules, and diabetes

In the questionnaire, participants were asked about their working hours at baseline using the following question: “During the last month, excluding summer vacations or a week containing many holidays, how many hours on average in a week did you work, including unpaid overtime work?” Participants reported their working hours as a continuous variable, and working hours were then categorized as 35–44 or ≥45 hours per week. We defined long working hours as ≥45 hours per week. Because the number of working hours may be affected by seasonal variations, we added the month in which participants underwent their annual health checkup at baseline as a covariate. Shift work schedules were also queried in the questionnaire (no shift work, or shift work with or without night shifts). Because there were few cases of shift work with no night shifts (*n* = 48), shift work schedules were categorized as non-shift work or shift work.

The incidence of diabetes was confirmed by FPG concentration ≥126 mg/dL and/or self-reported diagnosis of diabetes by a medical doctor based on the questionnaire completed at the annual health checkup after the baseline. FPG concentration was measured from a blood sample drawn from the antecubital vein of the seated participant, with minimal tourniquet use after a 12-hour fast, using the amperometric method (Arkray, Kyoto, Japan). We did not ask participants about the type of diabetes because they may have not remembered this information correctly. In Japan, type 2 diabetes is common, but the incidence of type 1 diabetes among adults is very low.^13^ The date of diabetes onset was defined as the intermediate date between the date of the health checkup in which the diabetes diagnosis was confirmed and the date of the previous checkup. From the questionnaire, we obtained information on family history of diabetes at baseline, which was defined as having or having had a first-degree relative with diabetes.

### Other data collection

Basic characteristics of participants were gathered, such as age, educational level (≤12 years or >12 years), marital status (married, others, or unknown), and job category (clerical workers, professional workers, manual workers, or fire fighters). For example, professional workers included doctors, nurses, pharmacists, physiotherapists, radiographers, and technicians. Manual workers included conductors, drivers, garbage workers, machine operators, mechanics, and school janitors. Body mass index (BMI) was calculated from data obtained at the baseline health checkup. Lifestyle habits were assessed, including smoking status (current smoker or not), drinking habits (every day, sometimes, or never), and exercise habits (≥2 times per week and ≥30 minutes per session^14^; yes, others, or unknown). Hypertension (yes or no) was defined as systolic blood pressure ≥140 mm Hg and/or diastolic blood pressure ≥90 mm Hg and/or self-reported treatment for hypertension at baseline. Blood pressure was measured using an automated sphygmomanometer (USM-700G Si; Ueda Avancer Corp., Tokyo, Japan) placed on the arm of a seated participant who had been seated for a few minutes before measurement.

### Statistical analysis

First, we showed descriptive statistics about the basic characteristics of the participants according to working hours by shift work schedules. Second, multivariate hazard ratios (HRs) and 95% confidence intervals (CIs) for developing diabetes associated with long working hours were calculated separately by shift work schedules using a Cox proportional model. Participants working 35–44 hours per week were used as the reference category. We constructed three models: model 1 was adjusted for basic information of diabetes, such as age, FPG concentration at baseline, family history of diabetes, and the month in which participants underwent their annual health checkup at baseline; model 2 was additionally adjusted for basic characteristics of participants, such as educational level, marital status, and job category; model 3 was additionally adjusted for BMI, smoking status, drinking habits, exercise habits, and hypertension at baseline. We then treated working hours as continuous variables and calculated HRs for the association between increasing working hours by 10 hours per week and developing diabetes. Finally, to assess potential effect modification by shift work, we further analyzed the interaction model, including working hours as categorical variables, shift work schedules, the multiplicative interaction term, and the same covariates as in model 3.

Two-tailed tests were used, and *P*-values below 0.05 were considered statistically significant. All statistical analyses were performed using JMP Pro 11.0.0 for Windows (SAS Institute Inc., Cary, NC, USA).

## RESULTS

The median follow-up period was 5.0 years for non-shift workers (*n* = 2371) and 4.9 years for shift workers (*n* = 824). During this period, 138 non-shift workers and 46 shift workers developed diabetes. The incidence of diabetes was 13.4 per 1000 person-years for non-shift workers and 12.6 per 1000 person-years for shift workers.

Table 1 shows the characteristics of non-shift and shift workers by working hours. Compared with those working 35–44 hours per week, both non-shift and shift workers working ≥45 hours per week were more likely to be young and firefighters and to have low FPG concentration at baseline and were less likely to be manual workers and to have daily or no drinking habits. In addition to these, non-shift workers working ≥45 hours per week were more likely to have high educational level and to be professional workers and were less likely to be current smokers and to have exercise habits and hypertension. Shift workers working ≥45 hours per week were more likely to be current smokers and were less likely to be professional workers.

**Table 1.** Baseline characteristics of male participants according to shift work schedules and working hours (*n* = 3195)

	Non-shift workers (*n* = 2371)				Shift workers (*n* = 824)			
	
Working hours	35–44 h/week		≥45 h/week		35–44 h/week		≥45 h/week	

	(*n* = 1435)		(*n* = 936)		(*n* = 327)		(*n* = 497)	

	*n*	%	*n*	%	*n*	%	*n*	%
Mean (SD) age	49.1	(6.3)	46.1	(6.4)	48.6	(6.2)	46.2	(6.5)
Mean (SD) FPG, mg/dL^a^	92.9	(10.0)	91.7	(9.2)	92.7	(9.8)	91.1	(8.8)
Family history of diabetes								
Yes	205	(14.3)	130	(13.9)	46	(14.1)	74	(14.9)
Educational level								
>12 years	734	(51.1)	524	(56.0)	51	(15.6)	91	(18.3)
Marital status^b^								
Married	1232	(85.8)	819	(87.5)	301	(92.0)	442	(88.9)
Others	202	(14.1)	117	(12.5)	26	(8.0)	55	(11.1)
Job category								
Clerical	622	(43.3)	402	(42.9)	13	(4.0)	34	(6.9)
Professional	474	(33.0)	373	(39.9)	43	(13.2)	37	(7.4)
Manual	312	(21.8)	75	(8.0)	160	(48.9)	176	(35.4)
Fire fighters	27	(1.9)	86	(9.2)	111	(33.9)	250	(50.3)
Mean (SD) BMI, kg/m^2^	23.7	(2.8)	23.8	(2.7)	24.1	(3.1)	24.1	(3.0)
Smoking status								
Current	684	(47.7)	407	(43.5)	165	(50.5)	300	(60.4)
Drinking habits								
Every day	638	(44.5)	398	(42.5)	100	(30.6)	112	(22.5)
Sometimes	564	(39.3)	422	(45.1)	169	(51.7)	317	(63.8)
Never	233	(16.2)	116	(12.4)	58	(17.7)	68	(13.7)
Exercise habits^b,c^								
Yes	337	(23.5)	180	(19.2)	127	(38.8)	188	(37.8)
Others	1087	(75.7)	750	(80.1)	198	(60.6)	305	(61.4)
Hypertension^d^								
Yes	208	(14.5)	106	(11.3)	46	(14.1)	67	(13.5)

BMI, body mass index; FPG, fasting plasma glucose; SD, standard deviation.

^a^FPG concentration at baseline.

^b^Total proportion does not add to 100% owing to missing data.

^c^Exercise habits refer to ≥2 times per week and ≥30 minutes per session.

^d^Hypertension is defined as systolic blood pressure ≥140 mm Hg and/or diastolic blood pressure ≥90 mm Hg and/or under treatment at baseline.

HRs for the incidence of diabetes associated with long working hours by shift work schedules are shown in Table 2. Non-shift workers working ≥45 hours per week had decreased HRs compared with those working 35–44 hours per week in all models, but these were not significant. HRs for each 10-hour increase in working hours per week were also decreased, and significant HRs were found in models 2 and 3 (HR 0.71; 95% CI, 0.49–0.99 for both models). On the other hand, shift workers working ≥45 hours per week had a significantly increased risk of diabetes compared with those working 35–44 hours per week in all models (HR 2.36; 95% CI, 1.22–4.79, HR 2.26; 95% CI, 1.17–4.57, and HR 2.43; 95% CI, 1.21–5.10 in models 1–3, respectively). HRs for each 10-hour increase in working hours per week were not significant among shift workers; however, HRs were greater than 1.0. Effect modification by shift work was significant (*P* for interaction = 0.02).

**Table 2.** Hazard ratios for the incidence of diabetes associated with long working hours by shift work schedules among all participants (non-shift workers *n* = 2371, shift workers *n* = 824)

Working Hours			Model 1^a^		Model 2^b^		Model 3^c^	
		
	Person-years	Case^d^	HR	(95% CI)	HR	(95% CI)	HR	(95% CI)
Non-shift workers								
35–44 h/week	6022	93	1.00		1.00		1.00	
≥45 h/week	4295	45	0.83	(0.56–1.20)	0.79	(0.53–1.15)	0.84	(0.57–1.24)
Per 10-hour increase^e^			0.74	(0.51–1.01)	0.71	(0.49–0.99)	0.71	(0.49–0.99)
Shift workers								
35–44 h/week	1412	17	1.00		1.00		1.00	
≥45 h/week	2245	29	2.36	(1.22–4.79)	2.26	(1.17–4.57)	2.43	(1.21–5.10)
Per 10-hour increase^e^			1.20	(0.91–1.55)	1.14	(0.85–1.49)	1.19	(0.88–1.59)

CI, confidence interval; HR, hazard ratio.

^a^Adjusted for age, fasting plasma glucose concentration at baseline, family history of diabetes (yes, no), and month in which participants underwent their annual health checkup at baseline.

^b^Model 1+ additionally adjusted for educational level (≤12 years, >12 years), marital status (married, others, unknown), and job category (clerical workers, professional workers, manual workers, fire fighters).

^c^Model 2+ additionally adjusted for body mass index, smoking status (current smoker or not), drinking habits (every day, sometimes, never), exercise habits (≥2 times per week and ≥30 minutes per session) (yes, others, unknown), and hypertension at baseline (systolic blood pressure ≥140 mm Hg and/or diastolic blood pressure ≥90 mm Hg and/or under treatment) (yes, no).

^d^Case refers to diabetes.

^e^HRs for the association between increasing working hours by 10 hours per week and developing diabetes.

Additionally, we carried out an analysis of non-clerical workers only (*n* = 2124) by shift work schedules, as shown in Table 3. We did this because the majority of shift workers were non-clerical workers. The results were relatively similar to those found in the analysis of all participants. In model 3, the HR for workers working ≥45 hours per week was 0.90 (95% CI, 0.52–1.52) among non-shift workers and 2.28 (95% CI, 1.13–4.82) among shift workers, compared with those working 35–44 hours per week.

**Table 3.** Hazard ratios for the incidence of diabetes associated with long working hours by shift work schedules among non-clerical workers (non-shift workers *n* = 1347, shift workers *n* = 777)

Working Hours			Model 1^a^		Model 2^b^		Model 3^c^	
		
	Person-years	Case^d^	HR	(95% CI)	HR	(95% CI)	HR	(95% CI)
Non-shift workers								
35–44 h/week	3360	54	1.00		1.00		1.00	
≥45 h/week	2467	26	0.75	(0.45–1.22)	0.80	(0.47–1.32)	0.90	(0.52–1.52)
Per 10-hour increase^e^			0.87	(0.55–1.26)	0.91	(0.58–1.30)	0.94	(0.58–1.40)
Shift workers								
35–44 h/week	1359	17	1.00		1.00		1.00	
≥45 h/week	2096	28	2.25	(1.16–4.57)	2.16	(1.10–4.39)	2.28	(1.13–4.82)
Per 10-hour increase^e^			1.18	(0.89–1.53)	1.12	(0.84–1.48)	1.16	(0.86–1.56)

CI, confidence interval; HR, hazard ratio.

^a^Adjusted for age, fasting plasma glucose concentration at baseline, family history of diabetes (yes, no), and month in which participants underwent their annual health checkup at baseline.

^b^Model 1+ additionally adjusted for educational level (≤12 years, >12 years), marital status (married, others, unknown), and job category (professional workers, manual workers, fire fighters).

^c^Model 2+ additionally adjusted for body mass index, smoking status (current smoker or not), drinking habits (every day, sometimes, never), exercise habits (≥2 times per week and ≥30 minutes per session) (yes, others, unknown), and hypertension at baseline (systolic blood pressure ≥140 mm Hg and/or diastolic blood pressure ≥90 mm Hg and/or under treatment) (yes, no).

^d^Case refers to diabetes.

^e^HRs for the association between increasing working hours by 10 hours per week and developing diabetes.

Based on the significant results among shift workers, we further divided the categories of long working hours into 45–54 (17 cases per 1417 person-years) and ≥55 (12 cases per 828 person-years) hours per week and then explored the association between long working hours and diabetes in the same way as in model 3. HRs were 2.55 (95% CI, 1.16–5.75) for shift workers working 45–54 hours per week and 2.26 (95% CI, 0.92–5.50) for those working ≥55 hours per week, compared with those working 35–44 hours per week.

## DISCUSSION

This is the first prospective cohort study to investigate the association between long working hours and diabetes separately by shift work schedules.

In this study, non-shift workers tended to show decreased risks of diabetes associated with long working hours, even when the analysis was restricted to non-clerical workers. Previous studies also indicated a significantly decreased risk of diabetes among non-shift workers with long working hours.^8^^,^^10^ Nakanishi et al performed a prospective cohort study and showed that non-shift workers working ≥11.0 hours per day had a significantly decreased risk of diabetes compared with those working <8.0 hours per day (relative risk 0.30).^8^ Kuwahara et al conducted a cross-sectional study and found that non-shift workers working 45–79 or 80–99 hours of overtime per month had significantly decreased risks compared with those working <45 hours of overtime per month (odds ratios 0.86 and 0.59, respectively).^10^ These results suggest a possible decreased risk of diabetes among non-shift workers who work long hours; however, additional cohort studies with large sample sizes are needed.

The potential mechanisms involved in the protective effects of developing diabetes associated with long working hours among non-shift workers are not clear. However, as argued by Nakanishi et al,^8^ the differences in 24-hour energy expenditure among non-shift workers in accordance with the length of working hours observed in their study may be related to the results. They found that the longer non-shift workers worked, the more highly they exhibited energy expenditure. Although we did not evaluate energy expenditure in this study, this might be one of the potential explanations for such findings.

To our knowledge, this is the first study to document that shift workers with long working hours had a significantly increased risk of diabetes. A previous cross-sectional study showed no association between long working hours and diabetes among shift workers.^10^ However, as previously mentioned, differences in study design, sample size, categories of working hours, covariates, and results shown by sex or not are considered potential reasons for inconsistency with previous reports. Because the present study is a prospective cohort study, the temporality is clearer than in a previous cross-sectional study.^10^

Potential explanations for developing diabetes among shift workers with long working hours can be inferred from previous studies. One of the important physiological problems associated with shift work schedules is that eating and sleeping phases are changed.^15^ Concerning eating, shift work schedules may lead to changes in daily eating patterns, such as irregular meal frequency and intake, especially among workers with long working hours. Both of these irregular eating patterns are considered to be associated with insulin resistance^16^^,^^17^ and may lead to the development of diabetes.

Regarding sleeping, Buxton et al indicated that prolonged sleep restriction with concurrent circadian disruptions, as may occur among shift workers, reduced the resting metabolic rate and caused a reduction in insulin secretion.^18^ Sleep restriction is reported to be significantly associated with increases in cortisol, interleukin-6, and tumor necrosis factor-α, and these changes may contribute to insulin resistance.^19^ Sleep deprivation is also associated with decreased leptin levels, increased ghrelin levels, and increased hunger and appetite.^20^ Moreover, the sympathetic nervous system may be activated by sleep deprivation, leading to increased blood pressure and heart rate,^21^ and hypertension is a known risk factor for diabetes.^22^ Additionally, long working hours aggravates sleep conditions,^1^ which may lead to the development of diabetes.

The third health risk is related to stress. Occupational stress, such as effort-reward imbalance (ERI), is pointed out to be more prevalent among shift workers than daytime workers,^23^ and long working hours is also associated with ERI.^24^ ERI is related to increased risk of diabetes.^25^^,^^26^ In addition to occupational stress, shift workers may experience more stress related to their family or social life than non-shift workers. Because shift work schedules can experience considerable disruption of family and social activities as many of these rhythms of the general population are oriented around the day^15^; moreover, this situation may be worsened by long working hours. It is known that stress is associated with concurrent activation of the hypothalamic-pituitary-adrenal axis,^27^ which may contribute to insulin resistance through the effects of glucocorticoids.

In this study, we observed a relatively high incidence of diabetes compared with that reported in two former Japanese cohort studies among men.^7^^,^^8^ The total incidence of diabetes in our study was 13.2 per 1000 person-years (13.4 per 1000 person-years among non-shift workers). This was higher than that reported in Kawakami’s study, in which 46.3% of subjects were shift workers (1.95 per 1000 person-years)^7^ and in Nakanishi’s study, which included only non-shift workers (9.1 per 1000 person-years).^8^ These two previous studies were conducted in 1984–1992^7^ and in 1994–1999,^8^ respectively, and our study was performed in 2003–2009. The secular trend of diabetes prevalence in Japan may be related to differences in the incidence of diabetes.

In addition, disparities in the definition of diabetes may have affected study results. Kawakami et al defined diabetes following the WHO criteria (1980).^7^ Participants were first screened for glucosuria at a routine health checkup, and then FPG concentration was measured in participants with glucosuria. Finally, participants with FPG concentration ≥110 mg/dL underwent a 75 g oral glucose tolerance test to determine the diagnosis of diabetes. Nakanishi et al defined diabetes following the American Diabetes Association 1997 criteria for epidemiological studies.^8^ The incidence of diabetes was confirmed by FPG concentration ≥126 mg/dL or use of hypoglycemic medication. Our definition of diabetes was similar to the latter, but instead of medication use, we used a diagnosis of diabetes by a medical doctor that was self-reported on our questionnaire, because some participants may have developed diabetes but chose not to undergo treatment. The definition of diabetes by Kawakami et al^7^ was stricter than that used here or by Nakanishi et al.^8^ Therefore, the incidence was comparatively low. In recent years, a recommendation to use HbA1c for diagnosis of diabetes was issued in a report by the International Expert Committee on diabetes.^28^ We did not use HbA1c for the diagnosis of diabetes in this study because only 26.0% of participants had HbA1c data at baseline. We consider that the definition of diabetes in epidemiological studies needs to be discussed and standardized for further studies.

There are several strengths in the present study. First, our study is a prospective cohort study design. The second strength is related to our approach for investigating the association between long working hours and incidence of diabetes. Following previous reports,^1^^,^^12^ we defined long working hours as ≥45 hours per week, conducted statistical analysis separately by shift work schedules, and defined the reference group as participants working 35–44 hours per week. Because of our clear definition of long working hours and exclusion of the influence of shift work, we believe these approaches contribute to clear interpretation of our results.

Nonetheless, our study has some limitations. First, the number of participants and the number of new onset of diabetes in our study were relatively small. This led to low statistical power, and there is a possibility that we could not find significant associations other than those obtained. Second, detailed information about shift work schedules, such as duration, types (eg, two-shift, three-shift), and frequency (eg, times per week) was not obtained. As a result, shift work schedules in this study may vary on these points, and small differences in shift work schedules among shift workers might affect the results. Third, data on working hours were self-reported and might, therefore, include some inaccuracies. This may lead to non-differential misclassification, and HRs may be attenuated toward 1.0. Nevertheless, this study found risks of diabetes associated with long working hours among shift workers. Fourth, the relatively low response rate in our study might lead to difficulty in generalizing the results. Information on non-responders could not be obtained, so we could not compare the characteristics of non-responders with those of responders. However, a total of 94.2% of the participants were followed up in our study, which strengthens our findings.

In conclusion, we prospectively investigated the association between long working hours and diabetes by shift work schedules among male civil servants in Japan. We found that long working hours increased the risk of incident diabetes among shift workers. Meanwhile, the results among non-shift workers suggest the possibility of decreased risk of diabetes associated with long working hours. It is important to take shift work schedules into consideration when implementing health care measures for the prevention of diabetes among male workers.

## ONLINE ONLY MATERIAL

Abstract in Japanese.

## References

[1] Bannai A, Tamakoshi A The association between long working hours and health: a systematic review of epidemiological evidence. Scand J Work Environ Health. 2014;40:5–18. 10.5271/sjweh.3388 24100465

[2] OECD (Organisation for Economic Co-operation and Development). StatExtracts, Average annual hours actually worked per worker [Internet]. Paris: The Organisation for Economic Co-operation and Development; [cited 2015 Sep 10]. Available from: http://stats.oecd.org/Index.aspx?DatasetCode=AVE_HRS.

[3] The International Labour Organization. Working time around the world [Internet]. Geneva: The International Labour Organization; [cited 2015 Sep 10]. Available from: http://www.ilo.org/wcmsp5/groups/public/---dgreports/---dcomm/---publ/documents/publication/wcms_104895.pdf.

[4] The International Labour Organization. ILOSTAT Database: Employment by sex and weekly hours actually worked (Thousands)-Japan [Internet]. Geneva: The International Labour Organization; [cited 2015 Sep 10]. Available from: http://www.ilo.org/ilostat/faces/help_home/data_by_country/country-details/indicator-details?country=JPN&subject=HOW&indicator=EMP_TEMP_SEX_HOW_NB&datasetCode=YI&collectionCode=YI&_afrLoop=2217127849820182#%40%3Findicator%3DEMP_TEMP_SEX_HOW_NB%26subject%3DHOW%26_afrLoop%3D2217127849820182%26datasetCode%3DYI%26collectionCode%3DYI%26country%3DJPN%26_adf.ctrl-state%3D4dbwqhe0m_271.

[5] Yugami K, Sasaki S Working styles and motivation in the public sector. Jpn J Labour Stud. 2013;637:4–19 (in Japanese).

[6] The International Diabetes Federation. IDF DIABETES ATLAS [Internet]. The International Diabetes Federation. [cited 2015 Sep 10]. Available from: http://www.idf.org/sites/default/files/EN_6E_Atlas_Full_0.pdf.

[7] Kawakami N, Araki S, Takatsuka N, Shimizu H, Ishibashi H Overtime, psychosocial working conditions, and occurrence of non-insulin dependent diabetes mellitus in Japanese men. J Epidemiol Community Health. 1999;53:359–63. 10.1136/jech.53.6.359 10396483 PMC1756890

[8] Nakanishi N, Nishina K, Yoshida H, Matsuo Y, Nagano K, Nakamura K, Hours of work and the risk of developing impaired fasting glucose or type 2 diabetes mellitus in Japanese male office workers. Occup Environ Med. 2001;58:569–74. 10.1136/oem.58.9.569 11511743 PMC1740182

[9] Kroenke CH, Spiegelman D, Manson J, Schernhammer ES, Colditz GA, Kawachi I Work characteristics and incidence of type 2 diabetes in women. Am J Epidemiol. 2007;165:175–83. 10.1093/aje/kwj355 17071844

[10] Kuwahara K, Imai T, Nishihara A, Nakagawa T, Yamamoto S, Honda T, Overtime work and prevalence of diabetes in Japanese employees: Japan epidemiology collaboration on occupational health study. PLoS One. 2014;9(5):e95732. doi:10.1371/journal.pone.0095732. eCollection 2014. 10.1371/journal.pone.0095732 24787995 PMC4006795

[11] Virtanen M, Kivimäki M Saved by the bell: does working too much increase the likelihood of depression? Expert Rev Neurother. 2012;12:497–9. 10.1586/ern.12.29 22550976

[12] Gan Y, Yang C, Tong X, Sun H, Cong Y, Yin X, Shift work and diabetes mellitus: a meta-analysis of observational studies. Occup Environ Med. 2015;72:72–8. 10.1136/oemed-2014-102150 25030030

[13] Morimoto A, Nishimura R, Tajima N Trends in the epidemiology of patients with diabetes in Japan. JMAJ. 2010;53:36–40.

[14] Ministry of Health, Labour and Welfare. The National Health and Nutrition Survey 2012 [Internet]. Japan: Ministry of Health, Labour and Welfare; [cited 2015 Sep 10]. Available from: http://www.mhlw.go.jp/file/04-Houdouhappyou-10904750-Kenkoukyoku-Gantaisakukenkouzoushinka/0000032813.pdf (in Japanese).

[15] Harrington J Health effects of shift work and extended hours of work. Occup Environ Med. 2001;58:68–72. 10.1136/oem.58.1.68

[16] Farshchi HR, Taylor MA, Macdonald IA Regular meal frequency creates more appropriate insulin sensitivity and lipid profiles compared with irregular meal frequency in healthy lean women. Eur J Clin Nutr. 2004;58:1071–7. 10.1038/sj.ejcn.1601935 15220950

[17] Sierra-Johnson J, Undén AL, Linestrand M, Rosell M, Sjogren P, Kolak M, Eating meals irregularly: a novel environmental risk factor for the metabolic syndrome. Obesity (Silver Spring). 2008;16:1302–7. 10.1038/oby.2008.203 18388902

[18] Buxton OM, Cain SW, O’Connor SP, Porter JH, Duffy JF, Wang W, Adverse metabolic consequences in humans of prolonged sleep restriction combined with circadian disruption. Sci Transl Med. 2012;4:129ra43. doi:10.1126/scitranslmed.3003200. 10.1126/scitranslmed.3003200 22496545 PMC3678519

[19] Ohkuma T, Fujii H, Iwase M, Kikuchi Y, Ogata S, Idewaki Y, Impact of sleep duration on obesity and the glycemic level in patients with type 2 diabetes: the Fukuoka Diabetes Registry. Diabetes Care. 2013;36:611–7. 10.2337/dc12-0904 23150286 PMC3579358

[20] Spiegel K, Tasali E, Penev P, Van Cauter E Brief communication: Sleep curtailment in healthy young men is associated with decreased leptin levels, elevated ghrelin levels, and increased hunger and appetite. Ann Intern Med. 2004;141:846–50. 10.7326/0003-4819-141-11-200412070-00008 15583226

[21] Lusardi P, Zoppi A, Preti P, Pesce RM, Piazza E, Fogari R Effects of insufficient sleep on blood pressure in hypertensive patients: a 24-h study. Am J Hypertens. 1999;12(1 Pt 1):63–8. 10.1016/S0895-7061(98)00200-3 10075386

[22] American Diabetes Association Screening for type 2 diabetes. Diabetes Care. 2003;26 Suppl 1:S21–4. 10.2337/diacare.26.2007.S21 12502615

[23] Peter R, Alfredsson L, Knutsson A, Siegrist J, Westerholm P Does a stressful psychosocial work environment mediate the effects of shift work on cardiovascular risk factors? Scand J Work Environ Health. 1999;25:376–81. 10.5271/sjweh.448 10505664

[24] Tsutsumi A, Kayaba K, Nagami M, Miki A, Kawano Y, Ohya Y, The effort-reward imbalance model: experience in Japanese working population. J Occup Health. 2002;44:398–407. 10.1539/joh.44.398

[25] Kumari M, Head J, Marmot M Prospective study of social and other risk factors for incidence of type 2 diabetes in the Whitehall II study. Arch Intern Med. 2004;164:1873–80. 10.1001/archinte.164.17.1873 15451762

[26] Li J, Jarczok MN, Loerbroks A, Schöllgen I, Siegrist J, Bosch JA, Work stress is associated with diabetes and prediabetes: cross-sectional results from the MIPH Industrial Cohort Studies. Int J Behav Med. 2013;20:495–503. 10.1007/s12529-012-9255-0 22915148

[27] Tsigos C, Chrousos GP Hypothalamic-pituitary-adrenal axis, neuroendocrine factors and stress. J Psychosom Res. 2002;53:865–71. 10.1016/S0022-3999(02)00429-4 12377295

[28] International Expert Committee International Expert Committee report on the role of the A1C assay in the diagnosis of diabetes. Diabetes Care. 2009;32:1327–34. 10.2337/dc09-9033 19502545 PMC2699715

