# Supplementary material for: The Risk of Developing Diabetes in Association With Long Working Hours Differs by Shift Work Schedules
Source: J Epidemiol. 2016 Sep 5;26(9):481–7. doi: 10.2188/jea.JE20150155 (PMC5008968; doi:10.2188/jea.JE20150155)
Supplement: Abstract in Japanese. [file je-26-481-s001.pdf]

長時間労働に関連した糖尿病発症リスクはシフト勤務により異なる

坂内聖[1]、吉岡英治[2]、西條泰明[2]、佐々木幸子[1]、岸玲子[3]、玉腰暁子[1]

[1] 北海道大学大学院医学研究科社会医学講座公衆衛生学分野
[2] 旭川医科大学健康科学講座地域保健疫学分野
[3] 北海道大学環境健康科学研究教育センター

【目的】長時間労働の糖尿病に与える影響は現在までのところ明らかではない。一方、シフト勤務は糖尿病のリスク増加に関連すると指摘されている。そこで本研究では日本の公務員を対象とし、長時間労働と糖尿病の発症の関連をシフト勤務別に検討することを目的とした。
【対象と方法】本研究は 2003 年 4 月から 2009 年 3 月にかけて行われた前向きコホート研究である。ベースライン時（2003 年 4 月から 2004 年 3 月）に健康診断を受けた 35 歳以上の男性公務員 3195 名(非シフト勤務者 2371 名、シフト勤務者 824 名)をシフト勤務別に解析した。労働時間は質問票で尋ね、週当たり 35-44 時間、45 時間以上に分類した。糖尿病の発症は年次の健康診断において、空腹時血糖 126 mg/dL 以上あるいは医師の診断（質問票にて自己申告）で確認した。Cox 比例ハザードモデルにて長時間労働に関連した糖尿病発症リスク（ハザード比[HR]と 95%信頼区間[95%CI]）を算出した。
【結果】追跡期間の中央値は非シフト勤務者で 5.0 年、シフト勤務者では 4.9 年であった。追跡期間中に非シフト勤務者では 138 名が、シフト勤務者では 46 名が糖尿病を発症した。多変量解析の結果、非シフト勤務者では週当たり労働時間 35-44 時間のものに比較して、45 時間以上のもので HR が低下した（HR 0.84 [95%CI 0.57-1.24])。一方シフト勤務者で週当たり 45 時間以上働くものでは週当たり 35-44 時間のものに比較して、有意に増加した糖尿病発症リスクを認めた（HR 2.43 [95%CI 1.21-5.10])。また全解析対象者のうち、シフト勤務者はそのほとんどが事務員以外の職種で構成されていたため、解析対象者を事務員以外のものに限定して解析したが同様の結果であった。
【考察】長時間労働に関連した糖尿病発症リスクは、シフト勤務の有無により異なった。

キーワード：コホート研究、産業衛生、性、シフト勤務、労働時間
